# Psychological, psychosocial and physical barriers preventing nature-based intervention participation in adults with mental health disorders: A scoping review

**DOI:** 10.1177/13591053241270410

**Published:** 2024-10-10

**Authors:** Mark W Burrell, Jo Barton, Gina Yannitell Reinhardt, Carly J Wood

**Affiliations:** University of Essex, UK

**Keywords:** barrier, mental health, nature-based intervention, NBI, physical, psychological, psychosocial

## Abstract

Nature-based interventions (NBIs) are becoming a common mental health care referral option; however, little is known about the barriers to participation. Research reveals a concentration of evidence on the practical barriers with a paucity of guidance on the personal barriers as experienced by service users. This review explores what is known on the psychological, psychosocial and physical barriers as disclosed by adult mental health service users and the various stakeholders involved in NBI. Nine of the 104 articles screened met the inclusion criteria. The review identified a total of 47 barriers in which the majority were standalone barriers unique to the individual article or participant that generated them. However, other barriers suggest a level of universality with the greatest array of barriers identified in the psychosocial category. The review highlights an urgent need for further research on the psychological, psychosocial and physical barriers to NBI participation.

## Background

Mental health disorders are a global concern (Rajabzadeh et al., 2021). In 2019, 970 million people globally were living with a mental health disorder (World Health Organization, 2024). The Global Burden of Disease (GBD) demonstrated that mental disorders were among the leading causes of burden worldwide (GBD 2019 Mental Disorders Collaborators, 2022) with epidemiological and economic estimates suggesting a global burden from the impact on human health and the associated economic cost (estimated in 2019) of around USD 5 trillion (Arias et al., 2022).

Social interventions are increasingly being used for the treatment of mental disorders. Whilst the term social intervention has not been clearly defined, it has been referred to as ‘an intervention that promotes interpersonal-level interaction, by targeting social capital and social support within groups or communities’ (Nagy and Moore, 2017). Nature-based interventions (NBIs) are one type of social intervention which involve any activity that takes place in the natural environment. Alongside socialisation (Wakefield et al., 2022), NBIs encompass multiple known therapeutic practises shown to improve mental health including interaction with nature (McMahan and Estes, 2015) and physical exercise (Chekroud et al., 2018). A recent systematic review and meta-analysis exploring the effectiveness of NBIs for adults with mental disorders revealed improvements in depression, anxiety and positive affect (Coventry et al., 2021). NBIs are used globally, with activities such as ‘forest bathing’ in Japan, South Korea, Poland, China and Taiwan (Wen et al., 2019), ‘mood walks’ in Canada (Morrison, 2017) and care farming in the Netherlands (Hassink et al., 2020). In the UK, the Government has invested £5.77 million into utilising NBIs for the treatment of mental disorders (Gov.UK, 2021).

Despite the positive impact of NBIs as a mental health intervention, there is a growing body of research into the barriers affecting NBIs such as clinical, organisational, practical and bureaucratic barriers. Such barriers include a lack of high-quality evidence and outcome measurement tools (Tambyah et al., 2022; Van den Berg, 2017), unclear referral pathways, increasing transparency about what NBIs offer, local availability of green spaces, funding issues, staffing challenges and health and safety concerns (Jepson et al., 2010; Robinson et al., 2020; Wong, 2020). There is however a lack of research on the personal barriers such as psychological (relating to a person’s mind or thoughts), psychosocial (concerning processes that are both social and psychological) and physical (concerning the qualities connected with a person’s body as opposed to their mind). These aspects could prevent referral, uptake and attendance of NBIs. Furthermore, there is a lack of personal barrier research from the perspective of the service user. For instance, researchers highlight the need to explore service users’ participatory behaviour (Skayni, 2022), any negative experiences from participation (Wilkie and Davinson, 2021), reasons behind dropouts (Wood et al., 2022) and racialised access to green space (Robinson et al., 2022). Other frameworks demonstrate that the processes relied upon within the NBI paradigm, that is, nature engagement and socialisation could themselves act as barriers to attendance. For instance, biophobia describes an aversion to nature (Ulrich, 1993) while social identity theory discusses how social processes can be detrimental to well-being (Tajfel and Turner, 1979).

Alongside the acknowledgement of this gap in the literature is the appreciation that the existing evidence is often limited due to biased sampling. For example, previous samples often included those that were more engaged in NBIs (Barley et al., 2012), and who chose to participate (Firby and Raine, 2021), with no data collected from those who failed to engage (Cuthbert et al., 2021; Harris, 2017). Given the commitment and investment in NBIs as a treatment for adults with mental health disorders, understanding the potential barriers that this population may encounter is essential to refining the intervention, increasing attendance and engagement, and reducing dropouts. This scoping review aims to identify what is currently known on the psychological, psychosocial and physical (PPP) barriers interrupting referral, uptake and attendance of NBIs in adults with mental health disorders. The review defines the term ‘barrier’ as follows: An impediment to desired outcome or growth psychologically, emotionally, socially, personally, professionally or spiritually. Barriers in this project are the concerns, anxieties, difficulties, issues, challenges or problems, that service users experience in relation to NBI participation. These barriers could be identified by service users themselves or other stakeholders.

## Method

### Inclusion and exclusion criteria

The scoping review was undertaken following the 22-item PRISMA checklist (PRISMA-ScR) for scoping reviews (Tricco et al., 2018). Inclusion and exclusion criteria were formulated using the population, concept, context method (Peters et al., 2020) with the population being adults with mental health disorders, the concept is psychological, psychosocial and physical barriers, and the context NBIs. Article inclusion criteria comprised (i) NBIs involving exposure to either green or blue environments; (ii) written in English; (iii) 1973 (founding of the American Therapeutic Horticulture Association) to the present; (iv) Adults aged 18–65). Non-PPP barriers such as clinical, organisational, practical and bureaucratic barriers were excluded.

### Article identification and selection

A systematic database search took place between 21st December 2022 and 6th January 2023 and included PubMed for life sciences and the health care system, Medline, Web of Science for psychology and social sciences, PsychInfo for psychological, behavioural and social science, CINAHL (covering nursing, allied health and social work), EThOS, Open Dissertations, PROQuest and Web of Science Conference Proceedings. Reference and citation searching was conducted by checking the reference and citation lists of relevant studies generated in the database search. Supplemental Table 1 details the search terms used in each database, including the use of both English and American spelling, phrase searching and MeSH headings (adapted to the parameters of each database). The expander option ‘apply equivalent subjects’ box was checked where available to include similar topics that may have made use of differing terminology not identified in the search string. The searches were carried out one concept at a time (1. Nature-based intervention, 2. Adult Mental Health, 3. Barriers), then combined in a fourth search to produce the final result.

**Figure 1.**
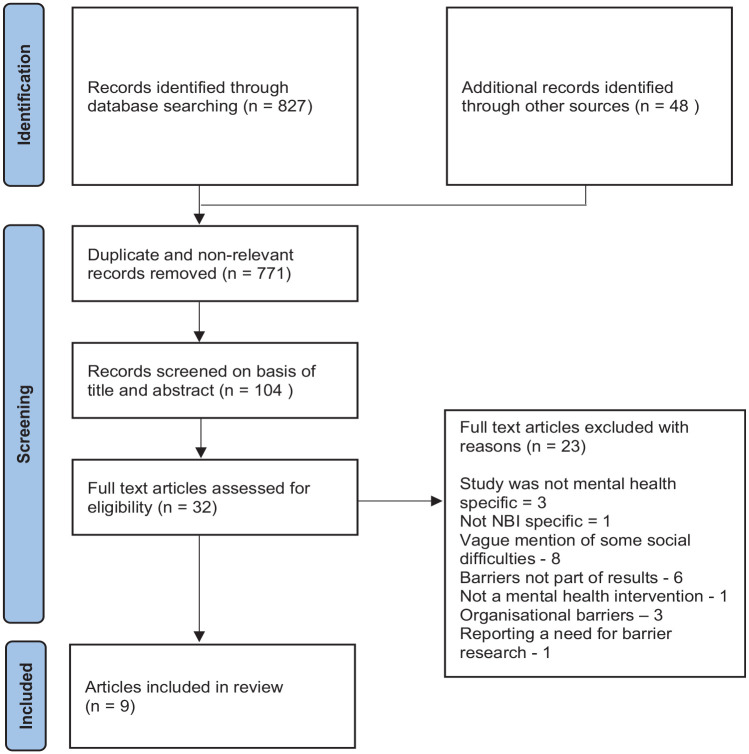
Preferred Reporting Items for Systematic reviews and Meta-Analysis (PRISMA) flowchart of study identification and selection (Tricco et al., 2018).

A total of 875 articles were identified. References were managed in EndNote 20 where non-relevant articles and duplicates were removed (*n* = 771). Initial selection of articles was based on title and abstract screening (*n* = 104) with articles read in full where title and abstract provided insufficient information. The full text review (*n* = 32) was carried out by the author and a second researcher independently with reasons for exclusion documented. Differences in application of inclusion/exclusion criteria were resolved by discussion. A total of nine articles were identified for inclusion (Figure 1).

### Data extraction

The extracted data was managed on a data charting tool based on the template by Peters et al. (2020). The data charting tool contained the key information of the source such as the author/s, date of publication, country of origin, population details, study design, intervention type and duration, and outcomes or findings that relate to the research question (i.e. PPP barriers). Data extraction of the final identified articles was charted by two reviewers independently with any inconsistencies discussed.

## Results

### Characteristics of sources of evidence

Across the nine studies, 167 stakeholders were interviewed. These were 85 NBI service users and 82 NBI facilitators. Facilitators included GPs, mental health clinicians, link workers, social workers, board members, managers, coordinators, students, researchers, volunteers, primary care mental health team members and ‘other service providers’. The data charting tool (Table 1) assigns study ID numbers to the nine articles which will be used throughout the results section when synthesising study characteristics and outcomes. The ID numbers also indicate whether the barriers were reported by service users (articles 1, 3, 4, 5, 7), stakeholders or clinicians (articles 2, 6, 8) or a mixed group (article 9).

**Table 1.** Data charting tool (adapted from Peters et al., 2020).

Study ID no.	Authors, date and location	Study design	Setting	Population	Intervention (duration)	Barrier outcomes and findings
1	Barley et al. (2012). London, England	Qualitative-semi-structured interviews	Primary care based horticultural and arts rehabilitation project, Sydenham garden	16 adult mental health service users referred to as co-workers. Nine male seven female. Age 38–91	Various attendance 2–4 years (*n* = 4) 1–2 years (*n* = 2) 3–12 months (*n* = 5) 6 weeks (*n* = 1)	Initial apprehension. Support needed to attend. Forced attendance. Participants claiming ownership, achievement comparisons. Resentment and exclusion feelings.
2	Fixsen and Barrett (2022). England and Scotland	Qualitative interviews via Skype, Teams, Telephone 30–60 minutes interviews	Four different NBI programmes in Scotland (*n* = 3) and England (*n* = 1)	Interviewees various stakeholders (*n* = 28) GP’s, link workers, managers, volunteers, researchers	No data given on any particular NBI service attendance or duration	Cultural expectations – to be prescribed medication. NBI not greeted with enthusiasm. Building trust with link workers. Stigma, social judgement on body ideals to outdoor recreation activities.
3	Harris (2017). England	Qualitative-Two Focus groups	1 acre walled garden run by a charity	Adult mental health service users with regular programme attendance (*n* = 15) Seven male eight female	Weekly gardening sessions up to 4 per week – open ended attendance. Average attendance 4.3 years	Deterioration in mental health (discussed as a temporary barrier) Lack of agency Lack of confidence to attend without a link worker.
4	Iancu et al. (2014) Netherlands	Qualitative-semi-structured interviews	13 care farms in two provinces in the Netherlands. 1 run by a mental health service, 12 owned privately	14 care farm service users. Age 18 years + (mean age 39.6 years). Predominantly Male	No data on any particular care farm attendance or duration	Mental health deterioration, tiredness, lack motivation. Social issues, peer relationships burdensome, arguments, not wanting to listen to others’ mental health problems.
5	Kyriakopoulos (2011). UK	Qualitative-semi-structured interviews. 35–45 minutes	A major UK university. Adventure Therapy (AT) location not given	Nine students/service users (only six interviews transcribed). White British, *F* = 3 age 20–22, *M* = 3 age 20–25	Combined one-to-one counselling (10–15 sessions) before AT, half day information and ice breaker session, 1 day of AT	Intense feelings of anxiety, fear of negative judgement, fear of not fitting in, isolation and rejection, fear of being a disappointment to others.
6	Marsh et al. (2018). Tasmania	Qualitative semi-structured face-to-face interviews (*n* = 16) and telephone interviews (*n* = 12) Interviews at a garden site (*n* = 16)	Community gardens in various locations. Veterans and families garden plot.	Interviewees (*n* = 26). Coordinators, volunteers, board members, managers, visiting social workers and other service providers	No data given on any particular NBI service attendance or duration	Volunteer attendance sporadic and volunteer relations were sometimes dysfunctional. Mental illness and lack of confidence, service users want someone to support them and to accompany them to the programme.
7	Sidenius et al. (2017). Horsholm, Denmark	Qualitative Semi-structured interviews approx. 20 minutes	Nacadia Therapy Garden	14 Nacadia service users with stress related diagnosis from ICD-10. Age 20–60	3 days per week, for 3 hours per day. Up to a maximum of 10 weeks	Initial start challenges – hesitant, anticipation. Social difficulties – how to interact with others, desire to withdraw socially, need to develop social coping strategies. Poor mental health – difficult to find a suitable location/activity.
8	Tambyah et al. (2022). Australia	Qualitative Semi-structured interviews	NBI within community mental health services, Illawarra	Mental health clinicians (*n* = 15) with a range or 2–40 years’ experience. Age 28–61. 80% female	No data on any particular NBI service attendance or duration	Mental health symptoms – lack of motivation, anxiety, panic attacks, socialising fears, social judgement, social comparison. Scepticism of NBI, GP buy-in, Medical model, no nature connection, physical limitations, lack of energy, not liking the activity.
9	Wood et al. (2022). Essex. UK	Qualitative Semi-structured interviews via zoom (*n* = 10) and telephone (*n* = 3) 20 minutes–1 hour 23 minutes and focus groups × 4. Total = 20 garden members. 30 minutes–1 hour.	Trust links mental health charity. Growing Together = 4 therapeutic garden sites.	Staff (*n* = 5), volunteers (*n* = 5), link workers (*n* = 2), primary care mental health team (*n* = 1). 20 service users (called members)	Attendance ranging from 4 months to 8 years	Unknown aspects of NBI. Meeting new people. Mental ill health. Changes to medication or failure to take medication. Upsetting other members and negative perception of social encounters. Physical health conditions, inability to engage, increased awareness of limitations.

Five studies reported on age which ranged from 20 to 91 years. Although the study reporting the age of 91 (1) was outside the age inclusion criteria, the study population concerned was adults in general. Four studies reported on gender (1, 3, 5, 8) with a total of 19 service users being male and 18 service users being female, and one study (8) reported their mental health clinician population as 80% female. The NBIs reported in the studies (where stated), were gardens; with four studies describing gardens run by charities (1, 3, 7, 9), one study describing community gardens (6), one with care farms (4) and one exploring adventure therapy (5). The remaining two studies did not report on the specifics of the NBI (8, 2). Intervention duration across the nine studies ranged from 6 weeks to 8 years, with one study reporting a single day (5). Three studies did not report on intervention duration (2, 6, 8). Frequency of intervention attendance data was only provided by two studies and included weekly sessions up to four times per week (3), and three sessions per week lasting 3 hours each (7).

All nine studies used qualitative methods, either semi-structured interviews or focus groups, although none of these studies specifically focused on the PPP barriers. Of the aims expressed across the nine studies, five explored the general views and experiences of service users (1, 3, 4, 5, 7) three explored NBI providers’ perceptions and responses to the implementation and function of NBIs (2, 6, 8) with only one of these specifically looking at the challenges (2), and one study explored the impact of NBIs on the mental health of service users along with a further aim of identifying barriers and facilitators (9). A total of 47 barriers were identified in which the majority were standalone barriers unique to the study that generated them (*n* = 36), with some barriers demonstrating possible universality (*n* = 11). A complete list of barriers with data extracts to illustrate the barriers are provided (Supplemental Table 4). Synthesis of results was conducted by reducing the 47 identified barriers down into 10 themed groups categorising similar concepts together (Table 2).

**Table 2.** Barrier groups by category (study ID numbers in parentheses).

Psychological group	Mental health	Referral	Activity		
Barriers	Mental health deterioration (3, 4, 6, 8, 9) Lack of confidence (3, 5, 6) Anxiety (5, 8) Medication (9) Lack of self-esteem (9)	NBI hesitancy (2, 7, 8, 9) GP buy-in (2, 8) Forced attendance (1) Loss of agency (3) Medical model expectation (2)	Lack of Motivation (4, 5, 8) Dislike of activity (8) Lack of nature connectedness (8)		
Psychosocial group	Social	Peers	Perception	Support	Public
Barriers	Social phobia (4, 5, 7, 8, 9) Judgement (2, 5, 8) Stigma (2, 8) Dwelling on patient identity (4) Development of coping strategy (7) Need to withdraw socially (7)	Not fitting in (5, 8) Achievement comparisons (1) Peer garden ownership (1) Resentment (1) Peer arguments (4) Burdensome peer relationships (4) Volunteer attendance (6) Volunteer relationships (6) Social Comparisons (8) Upset by peers (7)	Exclusion (1) Isolation and rejection (5) Disappointment to others (5) Upsetting others (9) Negative social encounters (9) Done wrong (9)	Supported Attendance (1, 3, 6) Initial apprehension (1) Link worker relationship (2) Additional intervention required (5)	Dislike public space (7, 8) Socially undesirable (8) Panic attack in public (8)
Physical group	Physical health	Energy			
	Poor physical health (8, 9) Inability to engage (9) Awareness of limitations (9)	Lack of energy (8) Tiredness (4)			

### Psychological barrier groups

The psychological barriers were catalogued into three distinct barrier groups labelled Mental Health, Referral and Activity. The Mental Health group contains five barriers of which deterioration in mental health was the most prevalent (3, 4, 6, 8, 9). Deterioration in mental health was reported as leading to dropouts or missed sessions, although one study commented that deterioration in mental health acted as a temporary barrier (3). Mental health symptoms and management of mental health were also referred to (8), with management reported as a barrier to engagement through changes to medication, and failure to take medication (9). Further psychological barriers that were identified by more than one study included anxiety and a lack of confidence. Lack of confidence was suggested as a reason for service users not attending without additional support (3, 6). Anxiety was mentioned as a potential barrier in two studies (5, 8) and was discussed as being linked to social phobia (8) and causing intense feelings of worry prior to starting the intervention at referral stage (5). This anxiety increased as the intervention start date got closer with all sources for the anxiety described as being related to the social aspects of NBIs. Finally, lack of self-esteem was identified by one study (9) and was described as occurring in service users when they first joined the programme.

The Referral group also contains five barriers. These are, forced attendance, loss of agency, GP buy-in, NBI hesitancy and medical model expectation. NBI hesitancy was the second most reported barrier across the psychological barrier groups (2, 7, 8, 9) and was linked to a level of scepticism, a lack of awareness of, and lack of belief in NBIs (8), anticipation of joining NBIs (2,7) and unknown aspects involved in NBIs (9). This barrier is bi-directional however, as study 2 reported that referral is influenced by the interest or lack thereof of the GP (GP Buy-in), and a patient’s expectations to be prescribed medicine. Thus, a patient could be reluctant to be prescribed NBI because they expect medication or because they detect a lack of enthusiasm from their mental health practitioner. Notable standalone barriers in the referral group of barriers include expectation of a medical model (2), loss of agency (3) and forced attendance (1). The forced attendance was only reported by one service user and was discussed not as a barrier to participation, but as a barrier to benefiting from the known health benefits of NBIs.

The Activity barrier group contains three barriers: lack of motivation, a dislike of the activity and not feeling connected to nature. Lack of motivation appears to be linked to different psychological facets including mental health deterioration (4, 8) and as a barrier in responding to the intervention that is, a disbelief in NBIs, and to NBI activities, with a particular dislike for physical activity (8). A lack of nature connectedness was the third barrier in this group and describes scepticism around the health benefits of engaging in nature. Clinicians have stated that some people are dismissive of the suggestion of spending time in nature. For instance, one clinician stated how their patient responded to a NBI referral by asking ‘Why? Why would you do that?’ (8)

### Psychosocial barrier groups

The largest amount of data collected was in the psychosocial barrier category and was represented across all nine studies. A total of 29 individual barriers were identified and grouped into five themed groups labelled Social, Peers, Perception, Support and Public. Within the Social group, the most frequently occurring barrier was that of a fear of socialising (4, 5, 7, 8, 9). The mechanisms behind why this barrier exists are unexplored, however, a fear of social judgement could be a contributing factor since it was the second most frequently reported barrier in this group (2, 5, 8). Another barrier in the Social group described in more than one study was stigma. Stigma was discussed as a result of low socioeconomic status; however, the research was located within socioeconomically deprived areas (2), and stigma was also attributed to association with a mental health group (8). Here clinicians suggested that stigma can arise from socialising in a mixed abilities group, or through feeling ashamed to be seen in public with a mental health group. A further explanation for stigma suggested that service users may not want to socially identify with people deemed unwell if they themselves did not identify with this label (8). The last three barriers catalogued in the Social group were all standalone barriers unique to the study that identified them. These were dwelling on patient identities with a dislike of hearing about others’ mental health problems (4), the need to develop coping strategies to deal with social interaction and the desire to withdraw socially (7).

The Peer group of barriers was the largest group (*n* = 10) however, all barriers were standalone barriers except one, ‘not fitting in’ (5, 8). Three of the barriers were reported in relation to peer relationships and included outcome comparisons with other service users over activity-based achievement, service users taking ownership over areas of the garden, and resentment towards other service users (as a result of the ownership; 1). Other Peer group barriers included, making social comparisons with other service users (8), concern from service users over whether group peers would cause them disturbances (7), arguments between peers, and peer relationships described as burdensome (4). Further to this, there were several concerns reported around volunteer relationships which suggested that volunteer attendance could be sporadic and that relationships with volunteers were sometimes dysfunctional (6).

The Support group contained four barriers of which only one barrier (supported attendance), was identified in multiple studies (1, 3, 6). The need for supported attendance was attributed to service users feeling too nervous to attend alone (1), a lack of confidence, and mental illness (3, 6). Supported attendance was discussed as a persistent barrier (3) with one interviewee stating that when the assisted attendance stops, service users do not have the confidence to return alone. Another form of support comes from the job role of the link worker, who is often the first point of call after referral. A suggested barrier here is that of failure to build a trusting relationship between service user and link worker (2). The third barrier in the Support group is initial apprehension and described how service users felt worried and chose not to attend unless persuaded to do so (1). The initial apprehension to attend was alleviated through being accompanied to the intervention (1), with supported attendance therefore being one way of overcoming this barrier. The fourth barrier listed is the need to have participated in counselling in order to facilitate attendance of a NBI.

All three barriers catalogued in the Public group were found in study (8), with one barrier (dislike public space) also occurring in study 7. Mental health clinicians in this study were concerned that being in a public space and the possibility of suffering a panic attack in public were potential barriers to service users with social phobia. One clinician described how a client of theirs was worried about people watching her and what they might be thinking of her. Furthermore, clinicians thought that group dynamics would present challenges to some service users. An example suggested was that younger participants may consider being grouped together with older people, or people less physically able, as socially undesirable.

The final themed group in the psychosocial groups is the Perception group. This group contains six potential barriers identified across three studies, and, as the group label suggests, are all perceived barriers existing in the minds of the service user. Perceived barriers included concerns over upsetting other service users, having a negative perception of social encounters, and believing in having done something wrong (9). The perception ‘having done wrong’ was reported by one service user who admitted that this type of negative belief would result in them not returning to the intervention (9). Further perceived barriers were concerns over being a disappointment to other service users, worrying over becoming isolated and rejected (5), and exclusion (1). Exclusion was perceived when things did not go the way a service user wanted it, when ideas put forward were not carried out, and not understanding why men could not attend a women’s only session.

### Physical barrier groups

The physical barriers were the least reported barriers only occurring in three studies. A total of five barriers were reported which were poor physical health, inability to engage, awareness of limitations, lack of energy and tiredness. These have been divided into two distinct groups, the Physical Health group, and the Energy group. Of these five barriers only one barrier (poor physical health), was reported in more than one study (8, 9). Physical activity was identified as exacerbating physical health problems, causing some form of suffering afterwards, with poor physical health resulting in an inability to engage fully in gardening tasks which led to an increased awareness of degenerative conditions (9). Physical limitations were also thought to contribute to a reluctance to join NBIs (8). The second physical barrier group (the Energy group) contained lack of energy, suggested by a mental health clinician (8), and tiredness (4). Although the origin of the reported tiredness is not transparent within the results, there is a suggestion that it may be linked to a deterioration in mental health.

## Discussion

This scoping review is the first to explore what is known about the PPP barriers experienced by adult service users of NBIs for mental health disorders. The psychosocial barriers were the most numerous (*n* = 29) followed by the psychological barriers (*n* = 13) then physical barriers (*n* = 5). Mental health, a fear of socialising and a disbelief in NBIs as a treatment for mental disorder were the most frequently identified individual barriers. The evidence identified in this review demonstrates a paucity of research on the PPP barriers with some studies obtaining data on barriers incidentally through explorations of the general experiences of service users as opposed to exploring barriers through a focused lens. The majority of data identified were standalone extracts unique to the study that identified them. This may be expected given the nature of qualitative research and the fact that such a small body of relevant research was identified. Despite this there were a range of barriers reported which included a number of commonly occurring barriers.

### Psychological barriers

The most frequently disclosed psychological barrier was a deterioration in mental health. Although the specifics of this barrier were not discussed, the deterioration could have an effect on other reported barriers such as a lack of confidence, motivation, self-esteem and could increase levels of anxiety, depression and social difficulties. Close behind the mental health barrier for frequency was a hesitation in NBI uptake. These findings are in line with the western medical model which emphasises the use of prescription medication (Fixsen and Barrett, 2022; McHale et al., 2020; Pescheny et al., 2018; Tambyah et al., 2022). An account of ‘GP buy-in’ further supports this where the success of the NBI referral is determined by the enthusiasm and personal belief of the mental health practitioner in order to positively encourage or influence the client (Fixsen and Barrett, 2022). However, while it is reported that people with mental disorder may lack motivation to attend such interventions, clinicians should remain conscious of a patient’s intrinsic motivation and past nature-based experiences. For example, intrinsic motivation to engage in nature is lower in people with mental disorders in comparison to the general population (Tester-Jones et al., 2020). Although social pressure does increase the likelihood of NBI engagement for people with mental disorder, it has also been linked with lower visit satisfaction and greater visit anxiety (Tester-Jones et al., 2020). Forced attendance was identified as a barrier in this review where one participant was made to feel obliged to attend a NBI whilst not feeling happy about doing so (Barley et al., 2012).

A further barrier that warrants discussion from the psychological category is a dislike for the activity. The activity itself acting as a barrier is important because NBIs are often assumed to be universally meaningful whilst ignoring the question of who the activity is meaningful for. One finding from the review questions the act of spending time in nature thereby suggesting evidence in support of the concept of biophobia. Biophobia (Ulrich, 1993) describes an aversion to nature and natural environments and suggests that humans have evolved with innate psychological responses to perceived threats found in nature, for instance spiders or snakes (Joye and De Block, 2011). Biophobia can also be present in people who have grown up in urban environments who may find an urban area more restorative than natural areas through processes of familiarity and material comfort, along with pleasurable experiences such as shopping or visiting cafes (Patuano, 2020). Caulkins et al. (2006) provide evidence for the occurrence of biophobia in NBIs with data highlighting how young women benefited less from hikes in the wilderness (in comparison to other participants), because of a high aversion to the outdoors. With the notion of biophobia in mind, NBIs should consider that not everyone will experience nature engagement as restorative and natural environments themselves can be a barrier to participation.

### Psychosocial barriers

Despite the reliance of NBIs on socialisation as a mechanism for improving wellbeing (Marsh et al., 2018), fear of socialising was the most commonly discussed psychosocial barrier. For instance, joining a NBI has been described as a daunting experience (Richardson et al., 2020) while getting to know people was deemed the greatest challenge of participating in NBIs (Sidenius et al., 2017). Indeed, becoming a member of a group in an NBI has been described as a major transition for service users, and one that can be experienced as exhausting (Nordh et al., 2009). It is possible that the daunting and challenging feelings expressed from service users manifest from being socially isolated or a lack of ability to socialise. For instance, research on a care farm in Norway reported that service users preferred to work with animals over being with people since interaction with people was deemed far too complex (Granerud and Eriksson, 2014). Furthermore, Ellingsen-Dalskau et al. (2016) reported that close social contact was described by one participant as ‘*out of the question*’. These findings reflect a recent report on green social prescribing where the most common attitudinal barrier identified was not wanting to take part in activities with unknown people (Gov.UK, 2023).

Another frequently mentioned barrier in the psychosocial category was the need to be accompanied to the NBI. This required support system was not necessarily a one-off occurrence to initiate first contact, it was suggested that for some, continued support was needed to maintain attendance (Harris, 2017). This finding supports discussions in the literature on the psychological distress experienced when entering a new social environment. For example, Millman and McNamara (2018) and Christie et al. (2008) reported on the experience of students starting university as bewildering, dislocating, involving alienation, exclusion, lack of belonging, low confidence and high self-doubt. If a non-mental health population can experience this level of distress, it is likely that a population diagnosed with mental health issues would experience a heightened version when entering a new social environment. For instance, the university student population who took part in a combined NBI with counselling study (Kyriakopoulos, 2011) reported that they would have been unable to take part in the NBI itself without first having undergone the counselling.

The concept of socialisation as causing harm is termed the social curse (Jetton, 2018; Kellezi and Reicher, 2012), and this review has identified data that supports this concept. Iancu et al. (2014) reported negative social encounters among NBI service users where peer relationships were described as burdensome, with accounts of arguments occurring between peers. There was also data that described a dislike of the tendency of individuals to dwell on patient identities. This extract is worthy of further investigation since it resonates with the research that demonstrates how some NBI service users like to distance themselves away from their patient identities and instead identify themselves as volunteers (Harris, 2017). Social identity theory (SIT; Tajfel and Turner, 1979) can be used to explain why dwelling on patient identities would upset some service users as it could prevent the successful identity transformation from a patient receiving health care, to perceiving oneself as a valued worker supporting a community garden. SIT postulates that people will want to remove themselves from a negative group identity and therefore being identified as a mental health service user, which is a stigmatised identity (Stuart, 2008), may be a potential barrier to NBI programme attendance.

### Physical barriers

The physical category contained the least barrier data. Wood et al. (2022) identified poor physical health, physical limitations and an awareness of one’s own limitations as barriers and highlighted how the physical difficulties to engage in activity led to an increased awareness of degenerative conditions. This is a significant finding which could have psychological implications such as increased depression through increased awareness of physical ill-health. For instance, Goldberg (2010) states that depression and chronic physical illness are reciprocal and suggests three distinct ways that chronic physical illness causes depression (which includes the suffering of pain and fear of disability). Furthermore, the increased awareness of a lack of ability could lead to social comparisons with other service users who are more capable, which could exacerbate certain mental health conditions further. This concept, however, is left unexplored and requires additional research to determine the psychological sequelae that could present with increased awareness of disabilities.

### Limitations

This review has a number of limitations. The participant population presents a limitation due to multiple factors. First, not all studies explored the experiences of the service users and instead relied on other stakeholders’ perceptions of what they thought would be barriers. Secondly, the service user population explored in the studies were able to maintain attendance and so the discussed barriers would have to be considered more speculative. Future research should try to access participants who dropped out of interventions to understand the barriers that led to this drop out. Furthermore, the barriers explored in this scoping review are specific to the age range dictated by the review question and therefore a different set of barriers could present for older adults and youth populations. The exclusion of non-English language could have limited the results; however, this was necessary due to time and resource limitations. The scoping review process is explorative in nature and due to this approach no attempts were made to assess the quality of the included studies. The absence of critical appraisal consequently results in an uncertainty around the quality of the results presented. A logical next step as more research in this area becomes available is to include a quality assessment. The strengths in this study are that it has highlighted an urgent need for further research into the PPP barriers and that it has demonstrated that the mechanisms described to be of therapeutic benefit to the service user that is, socialisation, nature, and physical activity, cannot necessarily be relied upon as being beneficial for all.

## Conclusions

In order to scale up NBIs for adult mental health care, it is of paramount importance that the barriers preventing referral, uptake and attendance are understood. This scoping review is the first to explore the PPP barriers experienced by adults with mental disorders attending NBIs. The key barriers identified in this review were deterioration in mental health, NBI hesitancy, lack of supported attendance, social phobia, social judgement and stigma surrounding mental health. The psychosocial barrier groups contained the most barriers and although NBI research has demonstrated that socialisation can induce psychological wellbeing, this review has demonstrated that facilitators of NBIs should be cautious of the socialisation element to act as a barrier. The review findings have highlighted an urgent need for further research in this area, with a specific focus on researching populations who have either refused referral or dropped out of a NBI.

## Supplemental Material

sj-docx-1-hpq-10.1177_13591053241270410 – Supplemental material for Psychological, psychosocial and physical barriers preventing nature-based intervention participation in adults with mental health disorders: A scoping review Supplemental material, sj-docx-1-hpq-10.1177_13591053241270410 for Psychological, psychosocial and physical barriers preventing nature-based intervention participation in adults with mental health disorders: A scoping review by Mark W Burrell, Jo Barton, Gina Yannitell Reinhardt and Carly J Wood in Journal of Health Psychology

sj-docx-2-hpq-10.1177_13591053241270410 – Supplemental material for Psychological, psychosocial and physical barriers preventing nature-based intervention participation in adults with mental health disorders: A scoping review Supplemental material, sj-docx-2-hpq-10.1177_13591053241270410 for Psychological, psychosocial and physical barriers preventing nature-based intervention participation in adults with mental health disorders: A scoping review by Mark W Burrell, Jo Barton, Gina Yannitell Reinhardt and Carly J Wood in Journal of Health Psychology
