# Supplementary material for: Psychological, psychosocial and physical barriers preventing nature-based intervention participation in adults with mental health disorders: A scoping review
Source: J Health Psychol. 2024 Oct 10;30(8):1735–50. doi: 10.1177/13591053241270410 (PMC12227812; doi:10.1177/13591053241270410)
Supplement: sj-docx-1-hpq-10.1177_13591053241270410 – Supplemental material for Psychological, psychosocial and physical barriers preventing nature-based intervention participation in adults with mental health disorders: A scoping review [file sj-docx-1-hpq-10.1177_13591053241270410.docx]

**Supplemental Files**

Table 1. Example search string used in each of the database searches.

| **Search** | **PubMed - Search String** | **Search Field** | **Results** | **Date** |
| --- | --- | --- | --- | --- |
| #1 | ("Gardening"[Mesh] OR "Horticultural Therapy"[Mesh]) OR ("Green social prescribing" [tw] OR "green prescri*" [tw] OR ecotherapy [tw] OR "community garden*" [tw] OR "care farm*" [tw] OR "nature-based intervention(s)" [tw] OR "nature-based progra*" [tw] OR "nature-based organisation(s)" [tw] OR "nature-based organization(s)" [tw] OR "nature-based health intervention(s)" [tw] OR "green care" [tw] OR "social therapeutic horticulture" [tw] OR "horticultural therapy" [tw] OR "peer outdoor exposure therapy" [tw] OR "green health partnerships" [tw] OR "green health referral" [tw] OR "green health prescription(s)" [tw]) | Text | 1586 | 05/01/2023 |
| #2 | (("Mental Disorders"[Mesh]) OR ( "Mental Health"[Mesh] OR "Community Mental Health Services"[Mesh] )) OR ("Mental health" [tw] OR "mental illness" [tw] OR "mental disorder" [tw] OR "mental ill-health" [tw] OR "poor mental health" [tw] OR "mental well-being" [tw] OR "mental wellbeing" [tw] OR "mental well being" [tw] OR "social isolation" [tw] OR "psychosocial well-being" [tw] OR "psychosocial wellbeing" [tw] OR "psychosocial well being" [tw] OR loneliness [tw]) | Text | 1,474,766 | 05/01/2023 |
| #3 | Barrier(s) [tw] OR obstacle(s) [tw] OR challenge(s) [tw] OR difficult* [tw] OR issue(s) [tw] OR accessibility [tw] OR negative(s) [tw] OR facilitator(s) [tw] OR enabler(s) [tw] OR psychological [tw] OR psychosocial [tw] OR physical [tw] | Text | 2,827,176 | 05/01/2023 |
| #4 | #1 AND #2 AND #3 | Text | 156 | 05/01/2023 |

Table 4. Barrier Definitions and Example Extracts.

| **Barrier (Alphabetical Order)** | **Description of Barrier** | **Example Extract from Article (ID in Parenthesis)** |
| --- | --- | --- |
| **Additional Intervention Required** | Having a need to undertake counselling prior to participation in a NBI | On the day I would have been a little bit too scared but the counselling kind of gave that little bit of extra push to go all the way. (5) |
| **Anxiety** | Feelings of excessive worry, fear, dread etc… | …this feeling of anxiety started to develop from the moment they accepted the referral to the outdoor transaction, gradually increasing in intensity as the event day drew closer… (5) |
| **Achievement Comparisons** | Comparing your accomplishments with others | …well there’s a lot more people in this garden, I’ll be honest, they’ve done a lot more work to what I’ve done… (1) |
| **Awareness of Limitations** | Partaking in activity highlights service users’ physical abilities | Sometimes it’s difficult when you want to do something and your physical problems mean you are going to suffer afterwards… (9) |
| **Burdensome peer relationships** | Socialising with other service users is a burden | Negative accounts of peer relationship were reported by the majority of participants on care farms. They described the relationships based on peer status as burdensome… (4) |
| **Development of Coping Strategy** | Developing a way to cope with participating in a NBI | …it took several participants some time to develop strategies to cope with the presence of others, and to get along with the other participants… (7) |
| **Disappointment to Others** | Worrying about not living up to other people’s expectations | …they feared … being a disappointment to others. (5) |
| **Dislike of Activity** | Not enjoying the activities available through the NBI | A lot of people just don't like exercise. (8) |
| **Dislike Public Space** | Not wanting to be participating in a NBI in public | The perception of being less exposed and being sheltered from the outer world in the fenced-off garden seemed to be an important part of creating a safe environment…(7) |
| **Done Wrong** | Believing you have made an error | … I sometimes think I’ve done wrong and it stops me coming again because I think to myself, I shouldn’t have done that…(9) |
| **Dwelling on Patient Identities** | A dislike of being identified as a mental health patient | …users were more likely to share personal problems, to constantly refer to situations related to mental disorders, and therefore to dwell on their patient roles. These participants avoided sharing their own problems with other users and disliked it when other users brought up their issues. (4) |
| **Exclusion** | Feeling like you are being left out or excluded | It’s a woman’s only group in the morning, which I felt a bit miffed about, because I felt it’s a community garden, I’m part of the community, why can’t I be here? (1) |
| **Forced Attendance** | Feeling like you are being persuaded or coerced to attend an NBI | I wasn’t 100% over happy about doing it. I felt like I was a bit obliged to do it, so I’m not happy about being obliged to do it… (1) |
| **GP Buy-In** | The clinician needs to be personally enthusiastic about NBIs in order to ‘sell’ it to the patient | Their willingness to recommend NBIs to consumers appeared to be linked with their personal connection with nature. (8) |
| **Inability to Engage** | Having limited ability to fully engage with the NBI activities | For one member, their inability to engage fully in gardening tasks and the recognition that their condition was degenerative gave them the ‘hump’. (9) |
| **Initial Apprehension** | Apprehension felt after referral or at the joining of a NBI | Several coworkers recalled initial apprehension: ‘I was a bit worried, but after a couple of weeks, I sort of settled in.’ (1) |
| **Isolation and Rejection** | Not being welcomed by other people or being left out of the group | …they feared becoming the recipient of other group members’ negative judgements, not fitting in, becoming isolated and rejected…(5) |
| **Judgement** | Worried about being judged by other service users | …she’s worried about people watching her while she’s walking and what they think of her. (8) |
| **Lack of Confidence** | Not feeling confident enough to attend or participate in a NBI | …without the prior counselling they would not even have considered participating in the outdoor event because they would have lacked the required confidence… (5) |
| **Lack of Energy** | Not feeling energetic enough to participate in a physical mental health intervention | Lack of motivation, lack of energy, lack of belief in the process… (8) |
| **Lack of Motivation** | A lack of desire or willingness to engage in a NBI | …clinicians claimed that lack of motivation when depressed…would be a major hindrance to participation. (8) |
| **Lack of Nature Connectedness** | Not feeling a connection to nature therefore not benefiting holistically from nature engagement | One clinician described her consumer’s response to a recommendation to spend more time in nature…the idea of going for a bushwalk just seemed quite weird to her like, ‘Why? Why would you do that? (8) |
| **Lack of Self-Esteem** | Being self-critical and feeling inferior to other people | Staff described how members are often lonely and isolated or lack confidence and self-esteem when they join. (9) |
| **Link Worker Relationship** | The Link Woker is a professional that makes up part of the clinical team in the green social prescribing pathway | The Link Worker was often a client’s first point of call, so building a trusting relationship was paramount… (2) |
| **Loss of Agency** | Inability to make decisions or have control over one’s life | …an episode of ill-health and accompanying loss of agency**…**may inhibit engagement in that moment. (3) |
| **Medical Model Expectation** | The expectation to receive pharmaceuticals or an evidence-based treatment option such as CBT | Patients’ notions about the role of the GP as there to prescribe medications were deeply entrenched in western society… (2) |
| **Medication** | Undesirable side effects from taking prescribed medication or failure to take medication | Staff and members described how declines in mental health, changes to medication or failure to take medication could cause members to disengage. (9) |
| **Mental Health Deterioration** | A period of poor mental health or decline in mental wellbeing | Reasons put forward centred upon personal explanations in relation to poor mental health emphasising the ups and downs characteristic of mental health distress: ‘I’m just trying to explain that it can be, it’s mental health… (3) |
| **NBI Hesitancy** | A reluctance to engage in a NBI or having a disbelief in the benefits of a NBI as a treatment for mental health disorders | Clinicians perceived that some consumers might not be willing to participate in NBIs due to scepticism and unawareness of its benefits. Clinicians highlighted that some people are oblivious to the natural environment and thus dismissive of its health and wellbeing benefits. (8) |
| **Need to Withdraw Socially** | A desire to remove oneself from social engagement | So, when I feel good, I choose something social and when I don’t feel so good, I choose something more withdrawn where I can be myself. (7) |
| **Negative Social Encounters** | Experiencing a social encounter in a negative way | …their mental health status negatively affected their perception of social encounters… (9) |
| **Not Fitting In** | Feeling out of place and unable to join in | … fear of having to socialize and/or not fitting into the group… (8) |
| **Panic Attack in Public** | Concern over people witnessing a panic attack episode | …consumers with anxiety and social phobia might feel uneasy about being in public spaces and concerned about a potential panic attack in front of others. (8) |
| **Poor Physical Health** | An impairment of normal physical function | …members with physical health conditions described how tasks could exacerbate issues… (9) |
| **Peer Arguments** | Arguments between service users of NBIs | … as peers, they argued… (4) |
| **Peer Garden Ownership** | Service users taking ownership over an area of a community garden | …the sense of ownership led to feelings of resentment when things did not go their way, such as: when their ideas had not been carried out; when, on one afternoon, men are excluded; or in tensions between groups over use of space… (1) |
| **Resentment** | A feeling of being treated unfairly | Cause as soon as I put [suggest] something, I get pulled back cause someone else has got it all. (1) |
| **Social Comparisons** | The evaluation of oneself or their abilities or traits | …sometimes, people, young people within that age group, the person who’s sort of presenting well is going to be like, “I’m not unwell like that person, I’m not coming”. (8) |
| **Social Phobia** | Having a fear of social situations | The most pronounced challenge that needed to be overcome was getting to know, and getting on with, the other participants… (7) |
| **Socially Undesirable** | Feeling embarrassed partaking in a social activity or behaviour. A concern over social status | …socially it would not be considered a “cool thing to do”. (8) |
| **Stigma** | A set of negative beliefs held by a group or society | Due to existing mental health stigma, some consumers could feel ashamed to walk with the group in a community setting. (8) |
| **Supported Attendance** | The need to have someone accompanying you in order to attend a NBI | To help with this nervousness in attending, some coworkers reported being accompanied, initially, by a healthcare worker. (1) |
| **Tiredness** | A decline in strength and energy | Several participants on both care farms and day centres referred to periods of deterioration, when they felt tired… (4) |
| **Upset by Peers** | Service users cause distress to others | Some were worried about the disturbances other participants might cause them… (7) |
| **Upsetting Others** | Concern over being a cause of distress to other service users | One member spoke of concerns around upsetting other members as a barrier to regularly attending… (9) |
| **Volunteer Attendance** | The reliance on volunteers to commit long term and help run NBIs | … some participants noted that volunteers were sporadic attendees …the reliance on volunteer labour was thought to hamper sustainable activities in some instances. Insufficient human resources or skills are an important barrier to being able to engage… (6) |
| **Volunteer Relationships** | The quality of relationships between service user and volunteers | … volunteer relations were sometimes dysfunctional. (6) |
